# Serum Reactivity Against Bacterial Pyruvate Dehydrogenase: Increasing the Specificity of Anti-Mitochondrial Antibodies for the Diagnosis of Primary Biliary Cirrhosis

**DOI:** 10.1080/17402520600668706

**Published:** 2006

**Authors:** Hiroshi Miyakawa, Atsushi Tanaka, Carlo Selmi, Naomi Hosoya, Norikazu Mataki, Kentaro Kikuchi, Takashi Kato, Junya Arai, Toshihiro Goto, M. Eric Gershwin

**Affiliations:** 1 Fourth Department of Internal Medicine Teikyo University School of Medicine Kanagawa 213-8507 Japan; 2 Department of Medicine Teikyo University School of Medicine Tokyo 173-8605 Japan; 3 Division of Internal Medicine, Department of Medicine Surgery and Dentistry San Pauro School of Medicine University of Milan Milan 359-8513 Italy; 4 Second Department of Internal Medicine National Defense Medical College Saitama Japan; 5 Division of Rheumatology Allergy and Clinical Immunology University of California at Davis School of Medicine Davis CA USA

## Abstract

Antimitochondrial antibodies (AMA) are the serum hallmark of primary biliary cirrhosis (PBC). However, AMA-positivity can be found in non-PBC sera when lower dilutions are used, thus raising issues about the specificity and sensitivity of the test. AMA reacts primarily with the lipoylated domains of pyruvate dehydrogenase-E2 (PDC-E2) which is highly conserved across species, including bacteria. We studied 77 serum samples, including 24 from patients with anti-PDC-E2-positive PBC and 53 controls (16 with autoimmune hepatitis (AIH), 10 with primary sclerosing cholangitis (PSC), and 27 healthy individuals) for their reactivities at serial dilutions (1:10, 1:20 and 1:40) against *Escherichia coli* DH5 alpha lysate overexpressing human PDC-E2 using immunoblotting (IB). A murine anti-human PDC-E2 monoclonal antibody (mAB) was used as control. We further studied positive sera using adsorption with a synthetic *E. coli* peptide sharing similarity with human PDC-E2. Finally, we verified whether a unique buffer for *E. coli* preparation could reduce non-specific serum reactivity. Results demonstrated that 100% of anti-PDC-E2-positive PBC and up to 38% of control sera at 1:10 dilution recognized *E. coli* PDC-E2 at IB while dilution tests indicated that the overall potency of PBC reactivity was 100-fold higher compared to controls. In fact, a subgroup (20-38%) of non-PBC sera were positive at low titers but lost the reactivity when absorbed with the synthetic *E. coli* peptide. Finally, our unique buffer reduced the reactivity of non-PBC sera as measured by ELISA. In conclusion, we demonstrated that weak cross-reactivity with *E. coli* PDC-E2 occurs in non-PBC sera at lower dilutions and that such reactivity is not due to AMA-positivity. The use of a specific buffer might avoid the risk of false positive AMA determinations when E. coli-expressed recombinant antigens are used.


*Clinical & Developmental Immunology*, June–December 2006; 13(2–4): 289–294

Taylor & Francis

Taylor & Francis Group

### Serum reactivity against bacterial pyruvate dehydrogenase: Increasing the specificity of anti-mitochondrial antibodies for the diagnosis of primary biliary cirrhosis

HIROSHI MIYAKAWA[1], ATSUSHI TANAKA[2], CARLO SELMI[3], NAOMI HOSOYA[1], NORIKAZU MATAKI[4], KENTARO KIKUCHI[1,5], TAKASHI KATO[1], JUNYA ARAI[1], TOSHIHIRO GOTO[1], & M. ERIC GERSHWIN[5]

[1]*Fourth Department of Internal Medicine, Teikyo University School of Medicine, Kanagawa 213-8507, Japan,* [2]*Department of Medicine, Teikyo University School of Medicine, Tokyo 173-8605, Japan,* [3]*Division of Internal Medicine, Department of Medicine, Surgery and Dentistry, San Pauro School of Medicine, University of Milan, Milan, Italy,* [4]*Second Department of Internal Medicine, National Defense Medical College, Saitama 359-8513, Japan, and* [5]*Division of Rheumatology, Allergy and Clinical Immunology, University of California at Davis, School of Medicine, Davis, CA, USA*

**Abstract**
Antimitochondrial antibodies (AMA) are the serum hallmark of primary biliary cirrhosis (PBC). However, AMA-positivity can be found in non-PBC sera when lower dilutions are used, thus raising issues about the specificity and sensitivity of the test. AMA reacts primarily with the lipoylated domains of pyruvate dehydrogenase-E2 (PDC-E2) which is highly conserved across species, including bacteria. We studied 77 serum samples, including 24 from patients with anti-PDC-E2-positive PBC and 53 controls (16 with autoimmune hepatitis (AIH), 10 with primary sclerosing cholangitis (PSC), and 27 healthy individuals) for their reactivities at serial dilutions (1:10, 1:20 and 1:40) against *Escherichia coli* DH5 alpha lysate overexpressing human PDC-E2 using immunoblotting (IB). A murine anti-human PDC-E2 monoclonal antibody (mAB) was used as control. We further studied positive sera using adsorption with a synthetic *E. coli* peptide sharing similarity with human PDC-E2. Finally, we verified whether a unique buffer for *E. coli* preparation could reduce non-specific serum reactivity. Results demonstrated that 100% of anti-PDC-E2-positive PBC and up to 38% of control sera at 1:10 dilution recognized *E. coli* PDC-E2 at IB while dilution tests indicated that the overall potency of PBC reactivity was 100-fold higher compared to controls. In fact, a subgroup (20–38%) of non-PBC sera were positive at low titers but lost the reactivity when absorbed with the synthetic *E. coli* peptide. Finally, our unique buffer reduced the reactivity of non-PBC sera as measured by ELISA. In conclusion, we demonstrated that weak cross-reactivity with *E. coli* PDC-E2 occurs in non-PBC sera at lower dilutions and that such reactivity is not due to AMA-positivity. The use of a specific buffer might avoid the risk of false positive AMA determinations when *E. coli*-expressed recombinant antigens are used.

**Keywords:** Escherichia coli, *autoantibodies, specificity, cross-reactivity*

**Abbreviations:** *AMA, antimitochondrial antibodies; PBC, primary biliary cirrhosis; AIH, autoimmune hepatitis; PSC, primary sclerosing cholangitis; NC, normal control; PDC-E2, pyruvate dehydrogenase complex-E2; IIF, indirect immunofluorescence; BHM, bovine heart mitochondria; SDS-PAGE, sodium dodecyl sulfate-polyacrylamide gel electrophoresis; mAb, monoclonal antibody; OD, optical density; E3BP, E3 binding protein; TLR, toll-like receptors*

High-titer antimitochondrial autoantibodies (AMA) are the serum hallmark of primary biliary cirrhosis (PBC) (Leung et al. 1997). The major AMA autoantigens belong to the 2-oxo dehydrogenase enzyme complex family with most patient sera reacting against the lipoylated domains of the pyruvate dehydrogenase complex-E2 (PDC-E2) (Gershwin et al. 1987; Coppel et al. 1988). Interestingly, the

Correspondence: M. E. Gershwin, Division of Rheumatology, Allergy and Clinical Immunology, University of California at Davis, Genome and Biomedical Sciences Facility, 451 E. Health Sciences Drive, Suite 6510, Davis, CA 95616, USA. Tel: 1 530 752 2884. Fax: 1 530 752 4669. E-mail: megershwin@ucdavis.edu

ISSN 1740-2522 print/ISSN 1740-2530 online © 2006 Taylor & Francis
DOI: 10.1080/17402520600668706

inner lipoylated domain of PDC-E2 is highly conserved across species (Fussey et al. 1990), thus suggesting that molecular mimicry by bacterial homologs might lead to AMA appearance (Van de Water et al. 2001). In fact, AMA cross-reactivity with non-human mitochondrial proteins such as *Escherichia coli* PDC-E2 occurs based on amino acid sequence similarity (Fussey et al. 1990). In the past years, new laboratory techniques have increased the sensitivity and specificity of AMA testing, particularly when recombinant mitochondrial antigens overexpressed in *E. coli* are used (Moteki et al. 1996; Miyakawa et al. 2001). In some cases, however, the use of recombinant antigens can potentially lead to AMA-positivity in non-PBC samples, particularly when lower serum dilutions are used, thus possibly limiting the specificity of the assay. Defining these non-PBC reactivities and finding a way to avoid false positive results appears critical to maximize the diagnostic power of AMA determination.

We hypothesized that non-PBC reactivities with lysates from *E. coli* overexpressing recombinant PDC-E2 might be secondary to low-affinity antibody binding to bacterial proteins, rather than the overexpressed human motif. We observed that reactivity against PDC-E2, either human or bacterial, could be found in a subgroup of non-PBC sera. However, reactivity was absent after adsorption of non-PBC sera with a bacterial synthetic peptide or following the use of a specific *E. coli* buffer, thus ultimately indicating novel ways to increase the sensitivity of AMA testing, recapitulating the extreme specificity of the AMA when human recombinant antigens are used.

### Materials and methods

### *Subjects*

Sera from non-PBC ($n = 53$) and PBC ($n = 24$) individuals were studied. Non-PBC sera included 16 patients with autoimmune hepatitis (AIH), 10 with primary sclerosing cholangitis (PSC), and 27 normal controls (NC). The diagnoses of AIH, PSC and PBC were based on internationally accepted criteria (Wiesner et al. 1985; Alvarez et al. 1999; Talwalkar and Lindor 2003). Briefly, all patients in the PBC group underwent liver biopsy and the diagnosis was made when two out of three criteria (i.e. presence of serum AMA $\geq$ 1:40, elevated serum alkaline phosphatase, and compatible liver histology) were met. Importantly, no histological features of other liver diseases (i.e. overlap syndromes) were observed in our PBC group. All sera were tested at serial dilutions for AMA using indirect immunofluorescence (IIF) on rodent tissue sections and then by immunoblotting (IB) using bovine heart mitochondria (BHM) as previously described.

### *Expression of recombinant PDC-E2 and preparation of* E. coli *lysate*

*E. coli* DH5 alpha was used as the vector to overexpress the recombinant human PDC-E2 protein (Moteki et al. 1996) and bacterial lysates were prepared as previously described (Eley et al. 1972). Briefly, plasmid DNA (PGEX-4T) was added to *E. coli* DH5 alpha competent cells, which were dissolved on ice and mixed. After heat shock treatment (42°C for 30 s), sterile SOC medium was added to the culture followed by shaking, and cells were then seeded on LB agar plate. After transformation, the cultured *E. coli* was rinsed, then frozen and fused several times, sonicated eight times for 30 s and centrifuged at 15,000 rpm to obtain the supernatant. The final protein concentration in the supernatant was 32.1 mg/dl as measured by Bradford Protein Assay (Bio-Rad, Hercules, CA, USA).

### *SDS-PAGE and immunoblotting*

The *E. coli* DH5 alpha lysates (100 µl/lane) were separated by sodium dodecyl sulfate-polyacrylamide gel electrophoresis (SDS-PAGE) (Laemmli 1970) and then transferred onto a polyvinylidene difluoride (PVDF) membrane. Sera were tested at 1:100, 1:1000 and 1:10,000 dilutions in PBS containing 0.1% Tween 20 as previously described (Towbin et al. 1979). Peroxydase-conjugated goat anti-human IgG, A and M (MBL, Nagoya, Japan) was used at 1:3000 dilutions as secondary antibody. The murine monoclonal antibody (mAb) C355.1 specific for the inner lipoylated domain of human PDC-E2 (Migliaccio et al. 1998, 2001) was used as positive control throughout the study. Known negative sera were also included in all experiments.

### *Preparation of the synthetic peptide for serum adsorption*

A comparison of PDC-E2 protein sequences in humans and from *E. coli* DH5 alpha indicated that two regions of human PDC-E2 (amino acids 36–49 and 163–176) include amino acid similarity with one region in the bacterial homolog (amino acids 31–44) (Figure 1); we then synthesized a peptide spanning *E. coli* PDC-E2 amino acids 31–44. The synthetic peptide was used at 1 mg/dl final concentration to

```

Human PDC-E2 (amino acid 36 - 49)                GDLIAEVETDKATV

                                                 *** ** ****

Human PDC-E2 (amino acid 163 - 176)              GDLLAEIETDKATI

                                                 * **

E.coli DH5 alpha PDC-E2 (amino acid 31 - 44)     EQSLITVEGDKASM

```

Figure 1. Amino acid sequence homology between human PDC-E2 and homologs from *E. coli* DH5 alpha. It is of note that the human sequences are also included in the triple expression hybrid pMIT3 that was used herein for AMA–ELISA.

Table I. AMA patterns of studied PBC and non-PBC sera analyzed by indirect IIF and IB with BHM. Anti-PDC-E2 positivity at IB was determined as the reactivity against the characteristic 74 kDa protein. Continuous variables are expressed as mean ± standard error.

| | Female sex (n) | Age (years) | AMA | | | | |
|---|---|---|---|---|---|---|---|
| | | | IIF | | | | 74 kDa* IB |
| | | | Negative | 1:10 | 1:20 | > 1:40 | |
| AIH ($n = 16$) | 14/16 | 56 ± 8 | 10 (63%) | 5 (31%) | 1 (6%) | 0 | 0 |
| PSC ($n = 10$) | 1/10 | 54 ± 7 | 8 (80%) | 2 (20%) | 0 | 0 | 0 |
| NC ($n = 27$) | 10/27 | 34 ± 8 | 20 (74%) | 7 (26%) | 0 | 0 | 0 |
| PBC ($n = 24$) | 21/24 | 56 ± 8 | – | – | – | 24 (100%) | 22 (92%) |

pre-incubate PBC and non-PBC sera that were previously shown to react with PDC-E2 at IB. After adsorption (overnight at 4°C) sera were tested against *E. coli* lysates at IB at equivalent concentrations.

*Use of a unique* E. coli *buffer in ELISA assay for AMA*

Serum samples ($n = 45$) from 20 patients with PBC and 25 non-PBC subjects were further studied at 1:1000 dilution for AMA using the triple expression hybrid clone pMIT3 as previously described (Miyakawa et al. 2001). Sera were tested using ELISA with and without the unique buffer (Miyakawa et al. 2001) and optical density (OD) values calculated and compared. Based on our previous data (Miyakawa et al. 2001), the OD value threshold for determining the AMA status was set at 0.130.

*Statistical analysis*

Group data were compared by the Welch's *t*-test and paired *t*-test. Statistical comparisons were made using SAS software (SAS Institute Inc., Cary, NC, USA). All analyses were two-sided, and $P$ values of $< 0.05$ were considered statistically significant.

### Results

*AMA*

Sera were tested for AMA using IIF at 1:10, 1:20 and 1:40 dilutions and IB against BHM at 1:100 dilution. Anti-PDC-E2-positivity at IB was considered as a reactivity against the characteristic 74 kDa band. Results of AMA testing are shown in Table I. Twenty four/24 (100%) PBC sera presented an AMA-specific IIF pattern at 1:40 while 22/24 (92%) were also anti-PDC-E2 positive at IB. Among non-PBC sera, 10/16 (63%) AIH, 8/10 (80%) PSC and 20/27 (74%) NC were negative for IIF–AMA at all dilutions or at IB. On the other hand, 6/16 (37%) AIH, 2/10 (20%) PSC and 7/27 (26%) NC were found positive for AMA–IIF at 1:10 dilution and negative at IB. Among these, one AIH serum was positive for IIF–AMA also at 1:20 dilution.

*Immunoreactivity against* E. coli *DH5 alpha lysate*

We investigated the immunoreactivity of sera against *E. coli* DH5 alpha lysate by IB. Sera were diluted at 1:100 in PBS and 24/24 (100%) PBC sera recognized one or both of two proteins of 74 and 52 kDa molecular weight. In particular, 23/24 PBC sera reacted with the 74 and 52 kDa proteins. Among control sera, 6/16 (38%) AIH, 2/10 (20%) PSC and 8/27 (30%) NC also reacted with either one or both proteins. It is of note that all but one NC non-PBC sera reacting with *E. coli* lysate were also positive for low titer AMA (≤ 1:20) by IIF. The results observed at IB are summarized in Table II and representative results for tested sera are shown in Figure 2B.

*Specificity of anti-PDC-E2*

The anti-human PDC-E2 C355.1 murine mAb was tested against the *E. coli* DH5 alpha lysate at IB for the definition of bands. As shown in Figure 2A, this antibody reacted with both 74 and 52 kDa proteins.

*Dilution tests*

Dilution tests were performed to compare the immunoreactivity of PBC and non-PBC sera at IB against *E. coli* DH5 alpha lysate overexpressing human PDC-E2. Test sera previously showing reactivity at 1:100 were serially diluted up to 1:10,000 in PBS. Twenty-two/24 (92%) PBC sera still reacted against the 74 and/or 52 kDa proteins at 1:10,000 dilution

Table II. Serial dilution analysis of PBC and non-PBC sera at IB for anti-PDC-E2 reactivity against *E. coli* DH5 alpha lysate.

| | Serum dilution | | | |
|---|---|---|---|---|
| | 1:100 | | 1:10,000 | |
| | 74 kDa (+) | 52 kDa (+) | 74- and/or 52 kDa (+) | |
| AIH ($n = 16$) | 6 (38%) | 6 (38%) | 6 (38%) | 1 (6%) |
| PSC ($n = 10$) | 1 (10%) | 1 (10%) | 2 (20%) | 0 |
| NC ($n = 27$) | 3 (11%) | 5 (19%) | 8 (30%) | 0 |
| PBC ($n = 24$) | 23 (96%) | 23 (96%) | 24 (100%) | 22 (92%) |

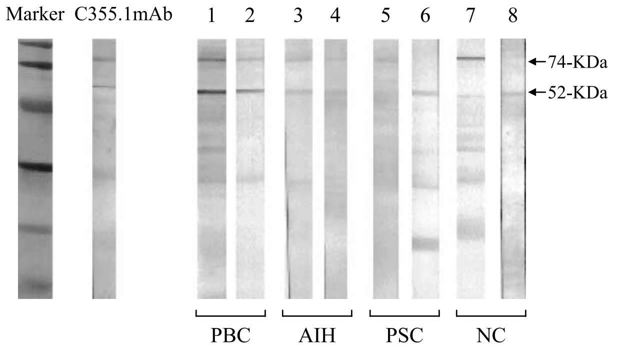

Figure 2. Representative IB pattern against *E. coli* DH5 alpha lysate overexpressing human PDC-E2 showing the 74 and 52 kDa reacting proteins. Results obtained with the murine anti-human PDC-E2 monoclonal antibody C355.1 (panel A), 2 anti-PDC-E2-positive PBC sera (lanes 1 and 2), 2 (AIH) sera (lanes 3 and 4), 2 PSC sera (lanes 5 and 6), and 2 NC sera (lanes 7 and 8) are depicted (panel B). Reactivities against the 74 (lanes 1–5 and 7) and 54 kDa (lanes 1–4, 6 and 8) proteins are shown.

whereas the reactivities of non-PBC sera were lost at 1:10,000 in all controls but 1/16 AIH sample (Table II). Two representative patterns of dilution tests for one PBC and one NC sera are shown in Figure 3.

### *Inhibition tests of* E. coli *DH5 alpha PDC-E2 synthetic peptide*

Following absorption with the synthetic bacterial peptide, 23/24 PBC sera still reacted against the 74 and/or 52 kDa proteins from the *E. coli* DH5 alpha lysate at 1:100 dilution. On the contrary, such reactivity was absent at the same dilution after adsorption in 15/16 (94%) previously positive non-PBC sera. The results of two representative adsorption experiments (one PBC and one NC sera) are shown in Figure 4.

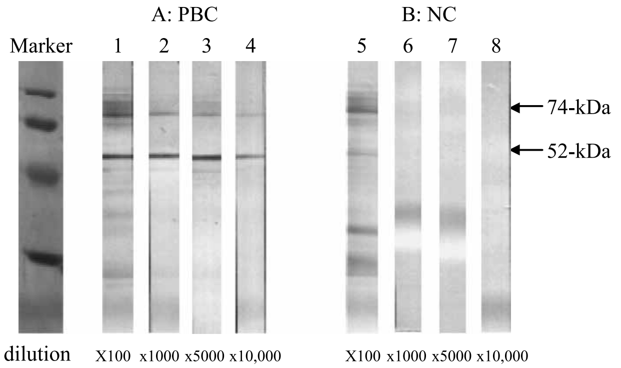

Figure 3. Dilution tests. Patterns observed with different serum dilutions of one PBC (panel A) and one NC serum (panel B) are shown. We note that the PBC serum reacts with the 74 and 52 kDa proteins at 1:10,000 dilution while reactivities were lost at 1:1000 for NC.

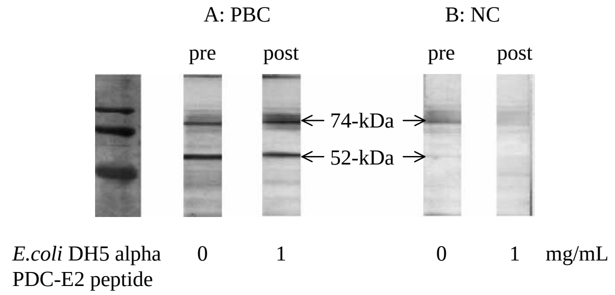

Figure 4. Adsorption tests. Sera were tested at IB after preincubation with the synthetic *E. coli* peptide. Reactivities with the 74 and 52 kDa proteins were visible after adsorption of the PBC serum (panel A) while reactivities were lost in the NC serum (panel B).

### *Efficacy of a unique* E. coli *buffer for AMA ELISA testing*

To investigate the effects of our unique *E. coli* buffer on ELISA assays for AMA testing we compared OD values obtained at ELISA with and without the buffer. Figure 5 illustrates the changes in OD values between the two conditions. PBC sera ($n = 20$) produced similar OD values when the buffer was added (2.187 ± 0.047 vs. 2.299 ± 0.046 without the buffer, $P =$ NS). Interestingly, when non-PBC sera ($n = 25$) were used, the addition of our *E. coli* buffer led to significantly lower OD values (0.031 ± 0.014 vs. 0.161 ± 0.005 without the buffer; $P < 0.001$). In 14 non-PBC sera, OD values obtained without the buffer were above the determined threshold and were therefore to be considered as AMA-positive. When our *E. coli* buffer was used, on the contrary, the decrease in OD values led to AMA-negativity in all 14 cases. Conversely, no change in AMA status was observed among PBC sera.

### Discussion

The accurate definition of serum AMA reactivities in patients with PBC is important for several reasons. First, despite the high sensitivity of AMA testing obtained with the use of *E. coli*-expressed recombinant antigens, the issue of its specificity remains, particularly when lower serum dilutions are used. Second, it is still debated whether PBC cases not presenting serum AMA are in fact lacking such autoantibodies or are secondary to limitations of the methods that do not detect low-titer AMA. Third, serum AMA specific for PDC-E2 appear to cross-react with bacterial mitochondrial antigens (4) while absorption of PBC sera with bacterial lysates fails to remove reactivities against mammalian antigens (16). These observations, alongside with the inhibitory effects of AMA on enzyme activity in different species (Teoh et al. 1994), are the bases of the mechanisms of molecular mimicry proposed in the pathogenesis of AMA and PBC (Van de Water et al. 2001). To confirm this

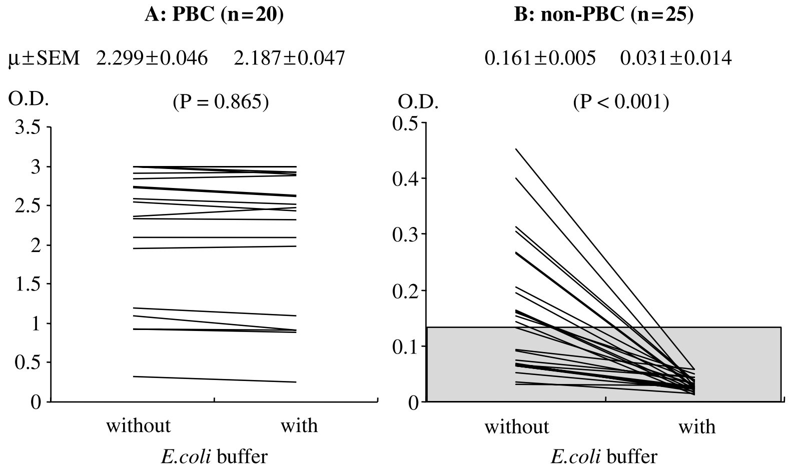

Figure 5. Comparison of OD values with and without our unique *E. coli* buffer for AMA–ELISA using the triple hybrid antigen pMIT3. Mean OD values were not different with the buffer for PBC sera ($n = 20$) while they were significantly lower when the buffer was used for non-PBC sera ($n = 25$). The OD threshold value (0.130) is represented in the right panel.

theory, therefore, it becomes critical to discriminate between cross-reactivities and independent humoral responses directed against similar proteins from different species, namely human and bacterial PDC-E2.

We report herein that AMA patterns detected when non-PBC sera are tested at IB with lysates of *E. coli* overexpressing recombinant human PDC-E2 are in fact due to reactivities against bacterial rather than human proteins. Conversely, reactivities of PBC sera are directed at human antigens. Moreover, we suggest that the use of our *E. coli* buffer can significantly increase the specificity of the technique for AMA testing while not reducing its sensitivity, thus suggesting novel ways to perform routine serum analysis.

We confirmed that PBC sera react with two proteins from *E. coli* DH5 alpha lysate. The nature of the bands of 74 and 52 kDa reacting with PBC sera at IB was defined using the validated murine anti-human PDC-E2 mAb C355.1 that reacted with both proteins. The definition of the band of lower molecular weight warrants further discussion since it could be mistakenly attributed to the cross-reacting E3 binding protein (E3BP, formerly protein X) (Dubel et al. 1999). Previous data from our group, however, demonstrated that C355.1 mAb does not cross-react with E3BP (Migliaccio et al. 1998); therefore, we considered that both bands corresponded to PDC-E2. We also report that 20–38% of non-PBC sera included in our study also reacted with both bands from the bacterial lysate when lower dilutions were used. Experiments based on serial serum dilutions demonstrated that non-PBC reactivity was 100-fold lower compared to PBC. Although based on limited evidence, low-titer AMA reactivity to mitochondrial antigens in non-PBC sera should be regarded as a relatively common finding, more often limited to IIF testing, in patients with autoimmune diseases other than PBC as well as in healthy subjects. To better define the nature of these weak reactivities we hypothesized that native bacterial rather than overexpressed human proteins might be the target of the antibody response in non-PBC sera. Data from our adsorption experiments using a synthetic *E. coli* peptide confirmed our working hypothesis. In fact, the IB pattern of PBC sera did not change after adsorption, while in all non-PBC sera but one from a patient with AIH reactivity was lost.

We then attempted to reduce ELISA non-specific antibody binding using our *E. coli* buffer. Interestingly, the addition of this buffer allowed a significant reduction in the reactivity of non-PBC sera while PBC reactivities were not affected. In particular, all 14 non-PBC sera presenting OD values exceeding the established threshold became AMA negative with the use of the *E. coli* buffer, thus proving to be most likely false AMA positive. The adsorption with the synthetic peptide might also be used to achieve similar results; however, an analysis of costs and effectiveness would easily prove that the use of our unique *E. coli* buffer is preferable to improve the specificity of AMA testing. In fact, ELISA is a well-established validated method for sensitive testing of AMA using recombinant antigens and should be regarded as a less expensive approach. Finally, we note that the hypotheses presented herein will benefit from additional studies on large populations of sera from patients with IIF–AMA-negative PBC or with recurrent urinary tract infections that will further define both sensitivity and specificity of the proposed AMA testing methods. However, we submit that our data have significant potential implications for AMA testing in clinical practice, by allowing higher sensitivity and specificity with the use of recombinant mitochondrial proteins rather than native proteins (Miyakawa et al. 2001).

Molecular mimicry by infectious agents (particularly bacteria) and xenobiotics might play a role in the initiation of the autoimmune reaction in PBC. Among bacteria, most evidence has been collected for *E. coli*, mostly based on the reported higher prevalence of recurrent urinary tract infections among patients with PBC (Gershwin et al. 2005). Additionally, microorganisms may contribute to autoimmunity via a polyclonal antibody activation and possibly the release of isolated antigens that could in turn activate the innate immune cascade. Bacterial molecules such as lipopolysaccharide (LPS) or CpG might then play a role as modulators of the immune response, as demonstrated by our group in serum hyper-IgM associated with PBC (Kikuchi et al. 2005), mediated by the binding to toll-like receptors (TLR) on macrophages and dendritic cells (Kaisho and Akira 2000, 2002). TLR might then activate innate immunity cells and stimulate the release of proinflammatory cytokines that in turn contribute to activate latent autoreactive T cells. The implications of our findings in this scenario are still largely unknown; however, we can submit some observations. First, we demonstrated that reactivity to bacterial mitochondrial antigens, albeit at low-titers, can be observed also in non-PBC sera, thus supporting the role of other infectious agents such as *Novosphingobium aromaticivorans* (Selmi et al. 2003). Second, we confirm that the occurrence of such AMA-like reactivity alone is unlikely to be sufficient at inducing PBC in healthy individuals. To initiate autoimmunity and perpetuate the immune-mediated tissue-specific injury that is PBC, in fact, additional immunological requirements must be met, most likely also based on a susceptible genetic background. As indicated by the high concordance in monozygotic twins (Selmi et al. 2004), in fact, the weight of genetic factors in determining susceptibility to PBC cannot be overlooked.

In conclusion, despite the lack of clinical association so far reported for AMA titers, we submit that an additional effort should be dedicated at definining the pathogenicity of these highly specific autoantibodies to unravel the enigmatic immunopathogenesis of PBC, possibly using animal models.

### References

Alvarez F, Berg PA, et al. 1999. International autoimmune hepatitis group report: Review of criteria for diagnosis of autoimmune hepatitis. J Hepatol 31:929–938.

Coppel RL, McNeilage LJ, et al. 1988. Primary structure of the human M2 mitochondrial autoantigen of primary biliary cirrhosis: Dihydrolipoamide acetyltransferase. Proc Natl Acad Sci USA 85:7317–7321.

Dubel L, Tanaka A, et al. 1999. Autoepitope mapping and reactivity of autoantibodies to the dihydrolipoamide dehydrogenase-binding protein (E3BP) and the glycine cleavage proteins in primary biliary cirrhosis. Hepatology 29:1013–1018.

Eley MH, Namihira G, et al. 1972. Keto acid dehydrogenase complexes. 18. Subunit composition of the *Escherichia coli* pyruvate dehydrogenase complex. Arch Biochem Biophys 152:655–669.

Fussey SP, Ali ST, et al. 1990. Reactivity of primary biliary cirrhosis sera with *Escherichia coli* dihydrolipoamide acetyltransferase (E2p): Characterization of the main immunogenic region. Proc Natl Acad Sci USA 87:3987–3991.

Gershwin ME, Mackay IR, et al. 1987. Identification and specificity of a cDNA encoding the 70 kd mitochondrial antigen recognized in primary biliary cirrhosis. J Immunol 138:3525–3531.

Gershwin ME, Selmi C, et al. 2005. Risk factors and comorbidities in primary biliary cirrhosis: A controlled interview-based study of 1032 patients. Hepatology 42:1194–1202.

Kaisho T, Akira S. 2000. Critical roles of toll-like receptors in host defense. Crit Rev Immunol 20:393–405.

Kaisho T, Akira S. 2002. Toll-like receptors as adjuvant receptors. Biochim Biophys Acta 1589:1–13.

Kikuchi K, Lian ZX, et al. 2005. Bacterial CpG induces hyper-IgM production in CD27(+) memory B cells in primary biliary cirrhosis. Gastroenterology 128:304–312.

Laemmli UK. 1970. Cleavage of structural proteins during the assembly of the head of bacteriophage T4. Nature 227:680–685.

Leung PS, Coppel RL, et al. 1997. Antimitochondrial antibodies in primary biliary cirrhosis. Semin Liver Dis 17:61–69.

Migliaccio C, Nishio A, et al. 1998. Monoclonal antibodies to mitochondrial E2 components define autoepitopes in primary biliary cirrhosis. J Immunol 161:5157–5163.

Migliaccio C, Van de Water J, et al. 2001. Heterogeneous response of antimitochondrial autoantibodies and bile duct apical staining monoclonal antibodies to pyruvate dehydrogenase complex E2: The molecule versus the mimic. Hepatology 33:792–801.

Miyakawa H, Tanaka A, et al. 2001. Detection of antimitochondrial autoantibodies in immunofluorescent AMA-negative patients with primary biliary cirrhosis using recombinant autoantigens. Hepatology 34:243–248.

Moteki S, Leung PS, et al. 1996. Use of a designer triple expression hybrid clone for three different lipoyl domain for the detection of antimitochondrial autoantibodies. Hepatology 24:97–103.

Selmi C, Balkwill DL, et al. 2003. Patients with primary biliary cirrhosis react against a ubiquitous xenobiotic-metabolizing bacterium. Hepatology 38:1250–1257.

Selmi C, Mayo MJ, et al. 2004. Primary biliary cirrhosis in monozygotic and dizygotic twins: Genetics, epigenetics and environment. Gastroenterology 127:485–492.

Talwalkar JA, Lindor KD. 2003. Primary biliary cirrhosis. Lancet 362:53–61.

Teoh KL, Mackay IR, et al. 1994. Enzyme inhibitory autoantibodies to pyruvate dehydrogenase complex in primary biliary cirrhosis differ for mammalian, yeast and bacterial enzymes: Implications for molecular mimicry. Hepatology 19:1029–1033.

Towbin H, Staehelin T, et al. 1979. Electrophoretic transfer of proteins from polyacrylamide gels to nitrocellulose sheets: Procedure and some applications. Proc Natl Acad Sci USA 76:4350–4354.

Van de Water J, Ishibashi H, et al. 2001. Molecular mimicry and primary biliary cirrhosis: Premises not promises. Hepatology 33:771–775.

Wiesner RH, LaRusso NF, et al. 1985. Comparison of the clinicopathologic features of primary sclerosing cholangitis and primary biliary cirrhosis. Gastroenterology 88:108–114.

